# Crossing the barrier or how regulation of ovastacin controls fertilization and translates into clinical phenotypes

**DOI:** 10.1016/j.isci.2025.112976

**Published:** 2025-07-01

**Authors:** Nele von Wiegen, Christian Behl, Hagen Körschgen

**Affiliations:** 1 Institute of Pathobiochemistry, University Medical Center of the Johannes Gutenberg University Mainz, Duesbergweg 6, D-55128 Mainz, Germany

**Keywords:** Biochemistry, Cell biology, Developmental biology

## Abstract

The *zona pellucida*, a glycoprotein matrix enveloping the mammalian egg, exerts essential functions during fertilization and early embryonic development. Its safeguard property regulates sperm entry and thus indirectly controls fertility. Limited proteolysis by the metalloproteinase ovastacin, released from the egg during fertilization, induces hardening of the *zona pellucida*. This precludes sperm entry and protects the embryo until implantation. However, ovastacin leakage before fertilization causes premature hardening and infertility if activity is not inhibited. This highlights the importance of ovastacin regulation by its endogenous inhibitor, fetuin-B. Accordingly, both loss and excessive ovastacin activity are linked to infertility. Here, we review recent discoveries on how ovastacin is precisely controlled to preserve *zona pellucida* permeability prior to fertilization and prevent penetration afterward. Based on these molecular mechanisms, we propose explanations for clinical phenotypes of recently discovered genetic mutations in ovastacin and discuss how modulation of ovastacin activity might be employed to regulate fertilization.

## Graphical abstract

**Figure:**
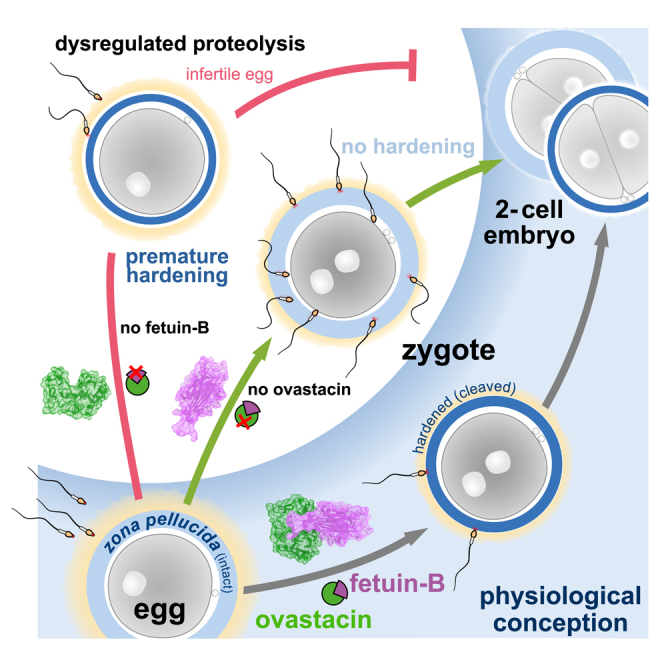

## Introduction

In the last decades, tremendous progress has been made toward our understanding of the molecular mechanisms orchestrating mammalian fertilization. Our current knowledge of this fundamental cellular interaction provides a functional explanation of numerous processes involved in gamete fusion, egg activation, and postfertilization modifications of the egg envelope, the *zona pellucida* (ZP). However, almost one in six individuals worldwide is affected by infertility,^1^ with up to 30% of cases being due to idiopathic causes,^2^ leading many to seek costly treatment options. This underlines the urgent need to elucidate the molecular mechanisms guiding fertility, including the regulation of fertilization, as the etiology of infertility remains unresolved for millions of affected people worldwide. A comprehensive understanding of the mechanisms involved might provide options to therapeutically modulate fertilization in a selective manner. This offers considerable potential not only to reduce the constantly rising financial costs of assisted reproductive technologies (ART)^3^^,^^4^ but also equally to reduce potentially harmful side effects for treated individuals.

The molecular mechanisms of specific aspects in egg-sperm interaction or the structures involved, such as the ZP, acrosome and cortical reaction, or the fertilization process itself, have been studied intensively (see reviews^5^^,^^6^^,^^7^^,^^8^^,^^9^^,^^10^^,^^11^^,^^12^^,^^13^^,^^14^). Despite considerable progress, certain gaps remain in our understanding of these processes. Among them, even though there are several candidates, a not-yet-identified ligand is responsible for binding of the sperm to the oocyte surrounding *zona pellucida* (ZP).^15^

Nevertheless, postfertilization cleavage of the ZP has long been recognized to induce a hardening of the ZP (ZPH), thus abrogating sperm binding and preventing sperm penetration. Recently, the field has gained valuable insights into how this ZPH is regulated by a precise modulation of the cortical granule proteinase ovastacin and disclosed how the ZP enables or prevents fertilization.^16^^,^^17^^,^^18^^,^^19^^,^^20^^,^^21^ These findings have uncovered the molecular basis of clinical pathologies that are associated with ZP modifications and uncovered therapeutic perspectives.

This review focuses on the mechanisms as well as the regulation of cleavage-induced ZPH and integrates these findings into clinically reported dysregulations. We concentrate on the mouse model, as the best studied in this context, and discuss the implications for humans based on clinical phenotypes. Observations in other species are explicitly indicated.

### A brief overview of mammalian egg-sperm interaction

**Figure 1:**
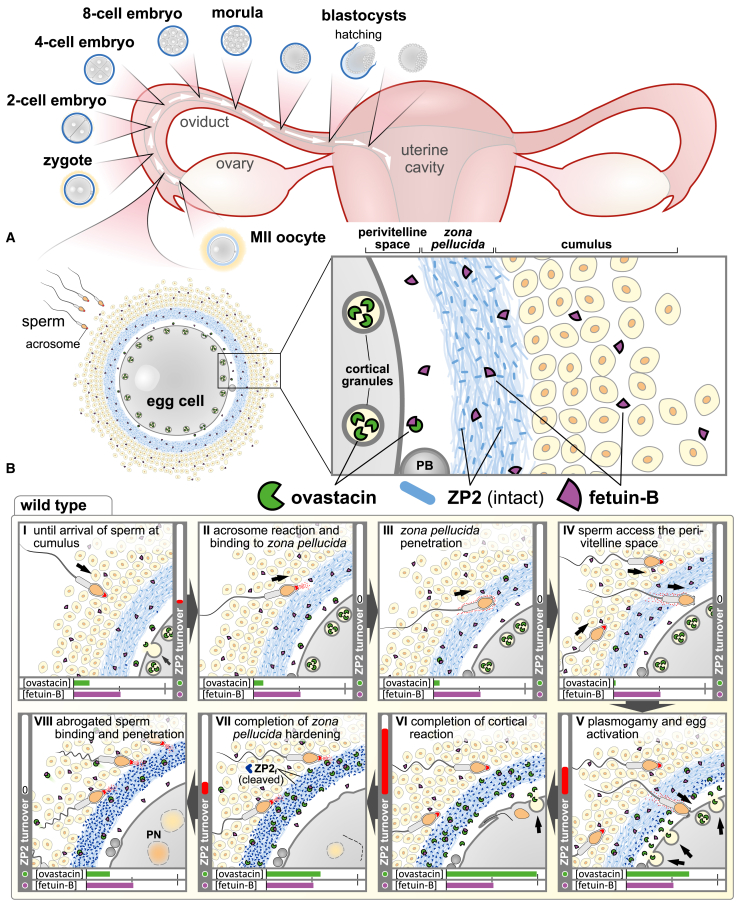
Schematic overview of mammalian egg-sperm interaction and fertilization focusing on the function of ovastacin and fetuin-B (A) Schematic overview of cumulus-oocyte complex before fertilization including detailed illustration of the barriers surrounding the egg in temporal and spatial context. (B) Chronological events of egg-sperm interaction (marked by arrow) starting with (I) arrival of sperm at the cumulus-oocyte complex, followed by (II) acrosome reaction and adhesion of sperm to the *zona pellucida* (ZP; light blue), (III) penetration of the ZP, (IV) sperm accessing the perivitelline space, (V) binding of the sperm to the oolemma and gamete fusion leading to exocytosis of cortical granules including release of the metalloproteinase ovastacin, and (VI) subsequent cleavage of ZP2 (dark blue angles). Thereby, ovastacin abolishes sperm binding to the ZP and hardens the ZP (dark blue) (VII and VIII). Horizontal bars approximate the respective concentration of ovastacin and fetuin-B in the vicinity of the ZP and the perivitelline space. The vertical bar (ZP2 turnover) approximates the temporal ZP2 conversion (relative ovastacin activity). Estimation of ovastacin release and regulation by fetuin-B based on the mouse model.^16^^,^^19^^,^^20^^,^^21^^,^^34^ Prematurely released ovastacin is blocked by fetuin-B to prevent premature ZPH; massive release of ovastacin as a consequence of the cortical reaction temporarily overcomes fetuin-B concentration, thus causing cleavage of ZP2 into ZP2_f_ and causing mechanical hardening of this extracellular matrix. Polar body (PB), pronuclei (PN).

Prior to mammalian fertilization and cortical reaction, which eventually induces ZPH, establishing an ultimate block against polyspermy, sperm have to overcome a set of selective barriers (Figure 1A). During their journey along the oviduct, before entering the cumulus oocyte complex (COC), spermatozoa undergo capacitation, thus obtaining their ability to fertilize the egg (see review^22^). In particular, this is characterized by the spermatozoan capability to exocytose their acrosomal vesicle upon reaching the cumulus, a layer of granulosa cells embedded in a hyaluronic acid matrix surrounding the egg, or at latest upon arrival at the ZP, the extracellular matrix of three to four glycoproteins (see “Biochemical, Structural and Phylogenetic Aspects” section) enclosing the egg (Figure 1B). The acrosomal reaction, among others, involves the release of hydrolytic enzymes such as hyaluronidase or the serine protease acrosin to assist sperm in their passage across the outer layers of the COC and thus leads to exposure of inner acrosomal membrane areas (see reviews^8^^,^^23^). However, the focal proteolysis of the ZP by acrosin is not mandatory in all species. In humans and hamsters, this selective ZP cleavage appears to be indispensable, as no penetration of the ZP and consequently no fertilization takes place without.^24^^,^^25^ In mice and rats, delayed penetration and fertilization is observed in the absence of acrosin activity.^26^^,^^27^^,^^28^ It is important to emphasize that the cleavage by acrosin is specific and limited rather than unspecific degradation.^29^^,^^30^ For additional ZP functions during fertilization, but also during oogenesis and preimplantation development, we refer to other reviews.^11^^,^^12^^,^^31^^,^^32^^,^^33^

Once the ZP has been passed successfully, the sperm enters the perivitelline space, the region between ZP and oolemma (Figure 1B). The interaction of IZUMO1, a transmembrane protein on the sperm surface, which is probably in a trimeric complex with TMEM81 and SPACA6, and oolemma Juno (also known as folate receptor 4) then mediates gamete fusion.^35^^,^^36^^,^^37^^,^^38^ This introduces, e.g., the phospholipase C zeta 1 (PLCz1) into the fertilized egg,^39^^,^^40^ triggering calcium oscillations that initiate egg activation and the cortical reaction (see reviews^5^^,^^7^^,^^41^). This affects the exocytosis of the content of regulated secretory vesicles in the periphery of the egg (termed “cortical granules”), whereby at least some of its contents appear to be trapped within the perivitelline space, thus creating a so-called “cortical granule envelope.”^42^ However, this release entails certain crucial events protecting the zygote from polyspermy and mechanical damage. For instance, (1) it induces shedding of Juno from the plasmalemma, thereby preventing further sperm fusion^35^^,^^40^ and (2) triggers release of the zinc-metalloproteinase ovastacin into the perivitelline space, where it cleaves *zona pellucida* protein 2 (ZP2) and converts it to its fertilized form ZP2_f_.^19^ This limited proteolysis triggers a conformational change of the ZP architecture, leading to its mechanical hardening (i.e., ZPH)^21^^,^^43^ and an abrogation of sperm binding^19^^,^^44^ (Figure 1B). Interestingly, this alteration of the ZP is strictly regulated. Overshooting ovastacin activity is associated with female infertility resulting from premature ZPH,^16^^,^^45^ whereas the lack of ZPH likewise affects a decline in fecundity or even leads to infertility.^20^^,^^46^ But how is this ovastacin-induced ZPH physiologically regulated and how does dysregulation affect fertility?

## Physiology of *zona pellucida* hardening and regulation of ZP2 cleavage

The egg-enveloping ZP, formed by cross-connected glycoprotein filaments (see “Biochemical, Structural and Phylogenetic Aspects” section), not only provides mechanical protection and offers a species-specific barrier to select the fittest sperm but also effectively functions as a gatekeeper. More than half a century ago, Braden et al. (1954)^47^ discovered the reaction of the mammalian ZP after fertilization that affects sperm binding and penetration by altering physicochemical properties of the ZP, resulting in ZPH.^48^ First in hamsters, these changes were soon attributed to components released from the cortical granules during the cortical reaction^49^ and initially assigned to the activity of a trypsin-like serine protease.^50^ Later, the target of this limited proteolysis was identified as ZP2.^51^ Jurrien Dean’s group^19^ was the first to uncover that not a serine proteinase, but a zinc-metalloproteinase termed ovastacin^52^^,^^53^ catalyzes this cleavage.

The fertilization-induced changes in the physicochemical characteristics of the ZP not only result in the abrogation of sperm binding and mechanical hardening but in some species also causes a thinning (in the murine ZP from approximately 8 to 6 μm thickness) and increases resistance to proteolytic degradation.^16^^,^^54^^,^^55^^,^^56^ We outlined major proteins associated with ZP’s barrier function in Table 1.

**Table 1:** Brief overview of major proteins associated with the modification of the *zona pellucida*’s barrier function

Protein name	UniProt acc. no.	Gene name	Ensembl acc. no.	Gene location chromosome	Protein location	Function	Reference
Acrosin	P23578	*Acr*	ENSMUSG00000022622	15	acrosome, secreted	limited ZP cleavage, mediate sperm penetration of the ZP	Hirose et al.^24^; Agarwal et al.^25^; Kuske et al.^30^; Urch et al.^57^; Mao and Yang^58^; Baba et al.^59^; Flörke-Gerloff et al.^60^
	P10323	*ACR*	ENSG00000100312	22			
Fetuin-B	Q9QXC1	*Fetub*	ENSMUSG00000022871	16	serum, follicular fluid	regulation of ovastacin activity	Dietzel et al.^16^; Cuppari et al.^17^; Karmilin et al.^61^; Pedersen^62^; Denecke et al.^63^
	Q9UGM5	*FETUB*	ENSG00000090512	3			
Ovastacin	Q6HA09	*Astl*	ENSMUSG00000050468	2	cortical granules, secreted into perivitelline space	ZP2 cleavage (mZP2166LA↓DE169, hZP2 171LA↓DD174), induction of ZPH	Burkart et al.^19^; Körschgen et al.^20^; Quesada et al.^52^; Quesada et al.^53^; Xiong et al.^64^
	Q6HA08	*ASTL*	ENSG00000188886	2			
Phospholipase C zeta 1 (PLCζ)	Q8K4D7	*Plcz1*	ENSMUSG00000030230	6	nucleus, perinuclear theca (sperm)	induction of intracellular Ca^2+^ oscillations in the egg, inducing egg activation and cortical granule exocytosis	Hachem et al.^39^; Nozawa et al.^40^; Malcuit et al.^41^
	Q86YW0	*PLCZ1*	ENSG00000139151	12			
*Zona pellucida* glycoprotein 1 (ZP1)	Q62005	*Zp1*	ENSMUSG00000024734	19	egg envelope	formation of ZP by crosslinking of ZP2/ZP3 heterodimer filaments	Burkart et al.^19^; Bokhove and Jovine^31^; Bleil et al.^44^; Bleil and Wassarman^65^; Rankin et al.^66^; Rankin et al.^67^; Rankin et al.^68^; Avella et al.^69^; Nishimura et al.^70^; Jimenez-Movilla and Dean^71^; Nishio et al.^72^; Wassarman and Litscher^73^; Miller et al.^74^; Greve and Wassarman^75^
	P60852	*ZP1*	ENSG00000149506	11			
*Zona pellucida* glycoprotein 2 (ZP2)	P20239	*Zp2*	ENSMUSG00000030911	7		formation of ZP, sperm receptor (binding to mZP2 aa^35^^,^^36^^,^^37^^,^^38^^,^^39^^,^^40^^,^^41^^,^^42^^,^^43^^,^^44^^,^^45^^,^^46^^,^^47^^,^^48^^,^^49^^,^^50^^,^^51^^,^^52^^,^^53^^,^^54^^,^^55^^,^^56^^,^^57^^,^^58^^,^^59^^,^^60^^,^^61^^,^^62^^,^^63^^,^^64^^,^^65^^,^^66^^,^^67^^,^^68^^,^^69^^,^^70^^,^^71^^,^^72^^,^^73^^,^^74^^,^^75^^,^^76^^,^^77^^,^^78^^,^^79^^,^^80^^,^^81^^,^^82^^,^^83^^,^^84^^,^^85^^,^^86^^,^^87^^,^^88^^,^^89^^,^^90^^,^^91^^,^^92^^,^^93^^,^^94^^,^^95^^,^^96^^,^^97^^,^^98^^,^^99^^,^^100^^,^^101^^,^^102^^,^^103^^,^^104^^,^^105^^,^^106^^,^^107^^,^^108^^,^^109^^,^^110^^,^^111^^,^^112^^,^^113^^,^^114^^,^^115^^,^^116^^,^^117^^,^^118^^,^^119^^,^^120^^,^^121^^,^^122^^,^^123^^,^^124^^,^^125^^,^^126^^,^^127^^,^^128^^,^^129^^,^^130^^,^^131^^,^^132^^,^^133^^,^^134^^,^^135^^,^^136^^,^^137^^,^^138^^,^^139^^,^^140^^,^^141^^,^^142^^,^^143^^,^^144^^,^^145^^,^^146^^,^^147^^,^^148^^,^^149^; hZP2, aa^39^^,^^40^^,^^41^^,^^42^^,^^43^^,^^44^^,^^45^^,^^46^^,^^47^^,^^48^^,^^49^^,^^50^^,^^51^^,^^52^^,^^53^^,^^54^^,^^55^^,^^56^^,^^57^^,^^58^^,^^59^^,^^60^^,^^61^^,^^62^^,^^63^^,^^64^^,^^65^^,^^66^^,^^67^^,^^68^^,^^69^^,^^70^^,^^71^^,^^72^^,^^73^^,^^74^^,^^75^^,^^76^^,^^77^^,^^78^^,^^79^^,^^80^^,^^81^^,^^82^^,^^83^^,^^84^^,^^85^^,^^86^^,^^87^^,^^88^^,^^89^^,^^90^^,^^91^^,^^92^^,^^93^^,^^94^^,^^95^^,^^96^^,^^97^^,^^98^^,^^99^^,^^100^^,^^101^^,^^102^^,^^103^^,^^104^^,^^105^^,^^106^^,^^107^^,^^108^^,^^109^^,^^110^^,^^111^^,^^112^^,^^113^^,^^114^^,^^115^^,^^116^^,^^117^^,^^118^^,^^119^^,^^120^^,^^121^^,^^122^^,^^123^^,^^124^^,^^125^^,^^126^^,^^127^^,^^128^^,^^129^^,^^130^^,^^131^^,^^132^^,^^133^^,^^134^^,^^135^^,^^136^^,^^137^^,^^138^^,^^139^^,^^140^^,^^141^^,^^142^^,^^143^^,^^144^^,^^145^^,^^146^^,^^147^^,^^148^^,^^149^^,^^150^^,^^151^^,^^152^^,^^153^^,^^154^), cleavage by ovastacin induces conformational change inducing ZPH	
	Q05996	*ZP2*	ENSG00000103310	16			
*Zona pellucida* glycoprotein 3 (ZP3)	P10761	*Zp3*	ENSMUSG00000004948	5		function for proper formation of ZP, proposed sperm binding	
	P21754	*ZP3*	ENSG00000188372	7			
*Zona pellucida* glycoprotein 4 (ZP4)	–	*Zp4*	ENSMUSG00000121372	13		function for proper formation of the ZP (pseudogene in mouse)	
	Q12836	*ZP4*	ENSG00000116996	1			

**Figure 2:**
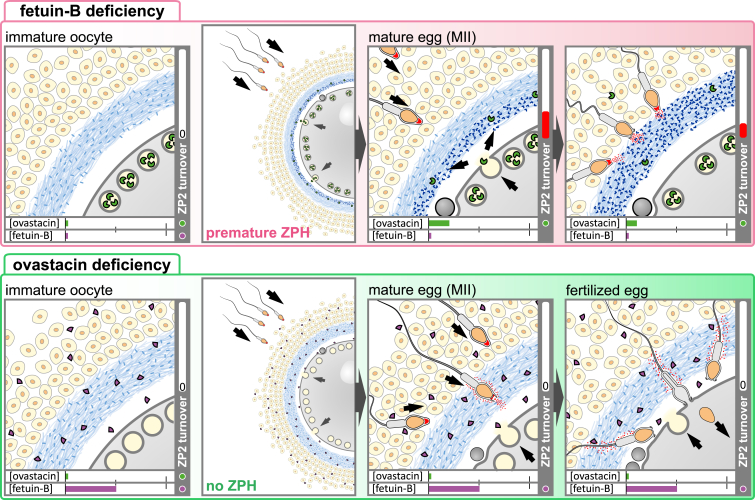
Schematic overview of pathophysiologic *zona pellucida* (ZP) reaction during egg-sperm interaction in fetuin-B-deficient (*Fetub*^−/−,^ upper row) or ovastacin-deficient (*Astl*^−/−^*,* lower row) mouse oocytes Chronological events during fertilization, starting with preovulation (germinal vesicle intact) oocytes (left). No inhibition of prematurely released ovastacin by fetuin-B causing premature conversion of ZP2 to ZP2_f_ (upper row right). This premature ZP hardening renders the ZP impassable for sperm and results in female infertility. Absence of ZP2 cleavage in ovastacin deficiency keeps the ZP passable for additional sperm even after fertilization without increasing the polyspermy rate *in vivo*. Horizontal bars approximate the respective concentration of ovastacin and fetuin-B in the vicinity of the ZP and the perivitelline space. The vertical bar (ZP2 turnover) approximates the temporal ZP2 conversion (relative ovastacin activity). Estimation of ovastacin release and regulation by fetuin-B based on the mouse model.^16^^,^^19^^,^^20^^,^^21^^,^^34^

Following the discovery of ZPH, additional postfertilization modifications besides ZP2 cleavage were identified. Here, we like to mention the deglycosylation of ZP3 and the “zinc sparks.” Sperm binding to ZP3 isolated from oocytes, but not to ZP3 from two-cell embryos, indicated ZP3 as the elusive primary sperm receptor at the ZP.^65^ This binding capacity had been attributed to *O*-linked glycosylation, which is lost after fertilization.^76^^,^^77^ However, successive deletion of ZP proteins in mice and their substitution by human orthologues revealed that sperm binding does not rely on ZP3 but on the cleavage state of ZP2.^66^^,^^67^^,^^68^^,^^78^^,^^79^ After the N-terminal region of ZP2 had been identified as the primary sperm receptor,^69^^,^^80^^,^^81^ the physiological implications of binding and postfertilization modification of ZP3 somewhat lost their significance of being essential, at least in these species. Nevertheless, *in vitro* the finding remains that sperm bind to both ZP2 and ZP3,^82^^,^^83^ which may trigger distinct processes. Sperm binding to the intact ZP therefore may also be more complex than monogenetic modifications currently indicate. Another exciting observation was “zinc sparks,” first reported even a decade ago.^84^^,^^85^ These sparks are described as secretions of free zinc (Zn^2+^) by the fertilized egg during activation and are believed to modify sperm binding and the physicochemical properties of the ZP.^86^ In a similar way to ovastacin, the magnitude of zinc release is believed to correlate with egg quality.^21^^,^^85^ So far, the “zinc sparks” have exclusively been detected *in vitro* via fluorescent zinc-selective chelating agents in culture media often containing chelators such as EDTA.^84^^,^^85^^,^^86^ However, depending on the affinity, these zinc-selective chelators may not exclusively bind free zinc. Accordingly, as these may influence the equilibrium of free and complexed zinc,^87^ a definite proof of free zinc is challenging. Interestingly, “zinc sparks” were not detected in ovastacin-deficient eggs and are substantially reduced in a zinc-deficient ovastacin mutant mouse model.^88^ Consequently, the physiological occurrence of “zinc sparks” *in vivo* as well as the functional implications remain elusive. These and additional postfertilization modifications of the ZP have already been extensively discussed by Fahrenkamp et al. (2020).^12^ In artiodactyls (even-toed ungulates), components of the oviductal fluid may also be involved in ZPH^89^^,^^90^^,^^91^^,^^92^; however this mechanism does not appear to be relevant in humans.^93^ Consequently, even though various postfertilization modifications have been reported, the literature so far indicates ovastacin-induced cleavage of ZP2 as most essential fertilization-regulating modification of the ZP, at least in mice and humans (Figure 2).^16^^,^^19^^,^^20^^,^^46^^,^^64^^,^^94^

Beyond the discovery of the causal relationships between ZP2 binding, ZP2 cleavage, and ZPH, the underlying mechanisms regulating these processes have now been uncovered. Although mouse models have clearly demonstrated that ovastacin is released during the cortical reaction to convert ZP2 into ZP2_f_ and induce ZPH (Figure 1B), they have also revealed the need for strict regulation. Dysregulated ovastacin activity, both excessive as well as reduced, is directly associated with infertility or at least reduced fecundity.^16^^,^^20^^,^^94^

*In vitro*, the redistribution and decrease in the number of cortical granules that coincides with the germinal vesicle breakdown in oocytes and extrusion of the first meiotic polar body during ovulation is associated with premature ZP2 cleavage.^16^^,^^95^^,^^96^^,^^97^ Given that this premature ZPH in mice and humans *in vitro* strongly affects fertilization success,^16^^,^^98^^,^^99^ it indicates that *in vivo* ZPH is not simply induced after but rather has to be prevented prior to fertilization (Figure 2)*. In vivo*, the small amount of ovastacin seeping from cortical granules prior to fertilization is controlled by its endogenous inhibitor, the plasma protein fetuin-B, to prevent premature ZPH-induced female infertility.^16^ This regulation is indispensable as ovastacin is stored in and released from the cortical granules in an active state.^20^ At present, neither autoactivation nor any activating proteases have been identified *in vivo* (see “Biochemical, Structural and Phylogenetic Aspects” section). Although physiological inhibition by fetuin-B occurs in the course of ovulation, it does not result in complete inhibition.^16^^,^^20^ The properties of the ZPs of wild-type, ovastacin-deficient, and double-deficient (i.e., ovastacin and fetuin-B) mice have been reported to be distinctly different.^20^^,^^21^ Prior to fertilization, these ZPs differ in the proportion of cleaved ZP2, resistance to proteolytic digestion, and mechanical properties (elastic modulus). This observation strongly points to cleavage of a minor proportion of ZP2 in mice *in vivo* even under physiological conditions. However, it is currently unknown whether and in which aspects these discernable properties have an impact on fertilization rate, polyspermy, or further development of the early embryo.

Various point mutations in ZP2 (e.g.,^100^^,^^101^^,^^102^) associate with developmental and implantation failure. Likewise, a lack of partial pre-fertilization cleavage of ZP2 might also influence hatching or early embryonic development. Complete absence of ovastacin-induced ZPH may be associated with premature hatching or reduced resistance to oviductal proteases, resulting in premature degradation of the ZP and embryo lysis.^103^ Although the mechanisms are not yet understood, the reduced fecundity of ovastacin-deficient mice, despite high monospermic fertilization success, and the infertility in humans carrying loss-of-function mutations in ovastacin indicate that at least a minimal ZP2 proteolysis is essential for successful development.^20^^,^^40^^,^^46^^,^^94^

Even though ZP2 cleavage provides a definitive block against polyspermy, there is emerging evidence indicating this block may not be the primary physiological function of ZP2 cleavage. Several studies did not detect polyspermy in ovastacin-deficient (*Astl*^−/−^) or ZP2-cleavage-impaired mouse models,^20^^,^^40^^,^^79^^,^^88^ and clinical mutations leading to loss of ovastacin activity (see “Clinical Significance” section) also fail to conclusively support the concept of loss-dependent polyspermy in humans. In the hamster, exocytosis of cortical granules is completed about 9 min after fertilization even after the formation of the primary polyspermy block.^104^ Additionally, conversion of ZP2 takes up to several hours.^19^^,^^40^ Consequently, these observations point against a primary function in polyspermy. Furthermore, *in vitro* fertilization (IVF) studies with a multiple surplus of sperm relative to the situation *in vivo* are not likely to elucidate the evolutionary function of ZP2 cleavage in polyspermy. However, other mutations affecting the architecture of the ZP, such as *Zp1*^−/−^ or *Zp2*^−/−^, while also not linked to polyspermy at physiological sperm concentrations, are associated with a reduced implantation rate.^68^ Considering the membrane block against polyspermy,^35^^,^^36^ which effects a rapid loss of the oolemma receptor Juno, a delayed ZP2 cleavage appears to be rather an additional backup block and more important for protection until implantation.

## Biochemical, structural, and phylogenetic aspects

In this section, we review biochemical insights into the proteins and structures involved in ZP2 cleavage in order to elaborate the molecular mechanisms underlying physiological ZPH induced by ovastacin and its regulation by fetuin-B.

### Ovastacin

**Figure 3:**
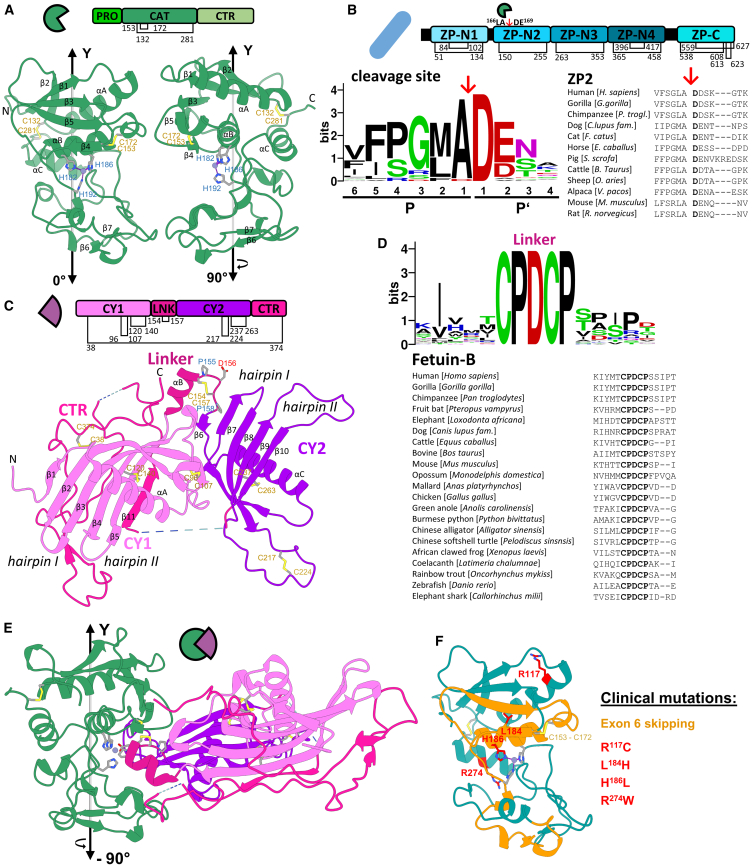
Structures of ovastacin and fetuin-B and conservation of the region in fetuin-B relevant for inhibition as well as of the cleavage site in ZP2 (A) Upper section: schematic domain structure of murine proovastacin (propeptide [PRO], catalytic domain [CAT], and C-terminal region [CTR]) including positions of disulfide bonds. Lower section: structure of the catalytic domain of murine ovastacin (AlphaFold3 model^128^) in standard orientation^129^ (left) and rotated 90° clockwise (right) depicting secondary structure elements (α-helices and β-strands), disulfide bonds labeled as yellow sticks, the histidine-coordinated catalytic zinc ion in magenta, and the termini. (B) Upper section: schematic domain structure of murine ZP2 including positions of disulfide bonds and indicating the ovastacin cleavage site. Lower section, left: sequence logo^130^ of the ovastacin cleavage sites of 30 mammalian ZP2 from different orders illustrates the total conservation of an aspartate residue in all mammals in position P1′. Positions in non-prime (P) and prime (P′) of the cleavage according to Schechter and Berger (1967).^131^ Lower section, right: sequence alignment of the cleavage site in ZP2 from selected mammalian species using ClustalX.^132^ (C) Upper section: schematic domain structure of murine fetuin-B (cystatin-like domain 1 [CY1], linker [LNK], cystatin-like domain 1 [CY1], and C-terminal region [CTR] including positions of disulfide bonds. Lower section: structure of murine fetuin-B (pdb 7AUW) depicting secondary structure elements (α helices and β-sheets), disulfide bonds as labeled yellow sticks and the termini. (D) Upper section: sequence logo^130^ of the linker of 38 vertebrate fetuin-B from different taxa illustrates the full conservation of CPDCP in all jawed vertebrates. Lower section: sequence alignment of the linker of fetuin-B from selected vertebrate with CPDCP in bold using ClustalX. (E) Structure of the complex of murine ovastacin (AlphaFold3 model) and fetuin-B (pdb 7UAW) in standard orientation rotated 90° counterclockwise. (F) Structure of the catalytic domain of human ovastacin (AlphaFold3 model) in standard orientation highlighting known clinical mutations and variants. Point mutations that cause a single amino acid exchange are marked in red; regions not translated as a result of a point mutation leading to a skipping of exon 6 are highlighted in orange.

In mammals, the ovarian-specific astacin or ovastacin is one of the six members of the astacin family of metalloproteinases.^52^^,^^105^^,^^106^ The astacin family also include the BMP-1/tolloid-like proteinases (BTPs) with BMP-1 (spice variant mTLD), mTLL-1, and mTLL-2, which are mainly involved in extracellular matrix (ECM) assembly and dorsoventral patterning in the early embryo.^107^^,^^108^^,^^109^^,^^110^^,^^111^^,^^112^ Further, it includes the meprins, α and β, both important in ECM remodeling and modulation of acute inflammation.^113^^,^^114^^,^^115^^,^^116^^,^^117^ Ovastacin, first described in 2004,^52^^,^^53^ displays the highest phylogenetic similarity to the hatching enzymes within the astacins. In non-mammalian vertebrates, these enzymes cleave the egg envelope to enable hatching of the embryo.^118^^,^^119^^,^^120^^,^^121^^,^^122^^,^^123^ Ovastacin is encoded by the *ASTL*/*Astl* gene, but it is phylogenetically not orthologous to other astacin-like (*astl)* proteins in non-mammalian species. In mice and humans, it is located on chromosome 2 and also known as SAS1B.^124^ Its oocyte-specific expression commences with the transition from primary to secondary follicle.^52^^,^^53^^,^^105^ Ovastacin accounts for approx. 1.5% of the total secretome of murine oocytes.^125^ The murine transcript is spliced out of nine exons^19^ and encodes a signal peptide (SIG 23 amino acids [aa], pos. 1–23), a propeptide (PRO, 66 aa, pos. 24–89) to ensure latency and localization within the cortical granules, the catalytic domain (CAT, 194 aa, pos. 90–283), and a C-terminal region (CTR, 152 aa, pos. 284–435) (Figure 3A). The human ortholog comprises 431 aa. In addition, several splice variants were identified,^124^^,^^126^ but their presence and function in oocytes is hardly understood. However, certain of these isoforms are expressed by distinct human cancer cells.^126^^,^^127^

Like all astacins, ovastacin is expressed as an inactive zymogen, proovastacin. Via the ^52^DKDIPAINQ^61^ sequence within the propeptide, ovastacin traffics through the endomembrane system to be stored peripherally in cortical granules.^64^

In homology to other astacins, the “aspartate-switch” mechanism, which utilizes D^77^ of the propeptide to interact with catalytic zinc ion, probably ensures latency of the zymogen.^133^^,^^134^^,^^135^ Unlike other members of this family, ovastacin is stored in an already partially activated form.^20^^,^^133^^,^^134^^,^^136^ However, this particular feature enables premature ZPH in the absence of fetuin-B^16^. The protease that activates ovastacin *in vivo* has yet to be identified*. In vitro*, ovastacin can be converted into the active form by trypsin-like serine proteases such as plasmin or acrosin.^30^^,^^61^^,^^137^

Like all representatives of the astacin family, named by its first described member “astacin,” a digestive enzyme from the gastric juice of the noble crayfish (*Astacus astacus*),^138^ ovastacin also contains the extended zinc-binding motif of the metzincins (^182^HExxHxxGxxH^192^).^139^^,^^140^^,^^141^ Furthermore, the catalytic domain features all structural elements typical for astacins (Figure 3A). The upper N-terminal subdomain comprises five conserved β-strands (β1-5) and three α-helices (αA-C). The conserved disulfide bonds are formed by C^132^-C^281^ and C^153^-C^172^. The lower C-terminal subdomain comprises two β-sheets (β6-7) and presumably two 3_10_-helices. Also conserved is the so-called “Met-turn” (SVM^236^HY), which is engaged in zinc binding and contributes to structural integrity.^142^ Accordingly, although the crystal structure has not yet been resolved, *in silico* predictions indicate ovastacin to feature the typical astacin-like fold. All mammalian ovastacins appear to fold identical.^18^ Moreover, the surface properties of their catalytic domain, which has sequence identity of ≥75%, involved in substrate interaction, also indicate a functional conservation of cleavage specificity.^18^

The specificity is characterized by the preference for acidic residues C-terminal to the cleavage site, which is typical for most astacins.^18^^,^^143^ This is also reflected in the cleavage site of the only known physiological substrate, ZP2 (in mice ^166^LA↓DE^169^)^19^ (Figure 3B). However, in contrast to other astacins with an acidic cleavage specificity, such as the meprins, ovastacin appears only to accept aspartate in position 1 on the prime side (nomenclature according to the concept of Schechter and Berger, 1967).^131^ Compared with the meprins, this is most likely based on a smaller substrate-binding pocket S1′, formed by R^264^, which sterically permits glutamate binding.^18^ Strikingly, with an aspartate at this position (P1′), the cleavage site is conserved in all mammalian ZP2 (Figure 3B). In general, ovastacin favors acidic residues in all positions of the prime side (P1′- P6′). We identified additional substrates with an attributed physiological function in the context of fertilization in the secretome of mouse embryonic fibroblasts.^18^ However, whether their cleavage indeed affects fertilization in a functional manner has yet to be identified. Currently, few details are known about the function of the CTR, which appears largely intrinsically disordered.^144^^,^^145^ Its phylogenetic origin is also still unknown.^52^ This region exhibits high variation in length with less than 50% sequence identity in mammals.^18^ In murine eggs, the CTR is cleaved off prior to secretion of ovastacin and appears to remain at the plasmalemma.^20^^,^^124^

### *Zona pellucida* and ZP2 cleavage

The egg envelope, i.e., the ECM surrounding the egg, fulfills a variety of functions, such as mediating species specificity, regulating fertilization, or protecting the early embryo from external hazards. Thus, this envelope (chorion/*zona radiata* in fish; perivitelline membrane in amphibians, reptiles and birds, or ZP in mammals) varies in function and properties according to the different ecological, fertilization, and developmental requirements within the different classes of vertebrates. However, what all species have in common is a modification of their egg envelope during fertilization and its composition comprising ZP proteins. Phylogenetically, vertebrate egg envelopes are encoded by ZP genes.^146^ Due to a rather confusing nomenclature throughout the literature, even within the mammals, as well as gene duplications and loss of ZP genes, interspecific comparisons regarding structure and effects of ZP modifications are challenging.^147^ In general, the mammalian ZP consists of four ZP proteins (ZP1, ZP2/ZPA, ZP3/ZPC, and ZP4/ZPB).^148^ For example, the human ZP consists of ZP1, ZP2, ZP3, and ZP4. Some species have lost ZP1 or ZP4; as in some mouse species, for example, ZP4 is pseudogenic and not expressed.^147^ For in-depth information on the phylogeny, function, and structure of the ZP, we refer to other reviews.^32^^,^^146^^,^^148^

All ZP proteins are highly glycosylated.^149^ However, in mouse ZP2, only one *N*-linked glycan is located close to the proposed sperm-binding region.^79^^,^^150^ Remarkably, the number and position of cysteine residues is conserved in mammalian ZP proteins and has been verified by X-ray crystallography.^150^^,^^151^^,^^152^^,^^153^ Both, the signal peptide and the transmembrane domain including the C-terminal tail are cleaved by furin during maturation. This causes detachment of the ectodomain from the oolemma, eventually resulting in the mature ZP protein.^150^^,^^154^^,^^155^^,^^156^ In their mature form, all mammalian ZP proteins comprise a C-terminal ZP module that is composed of a ZP-N and ZP-C sub-domain connected by a linker sequence. Apart from ZP3, the other ZP proteins feature additional ZP-N sub-domains N-terminal to the ZP module. For instance, mammalian ZP2s contain three of these (ZP-N1, ZP-N2, and ZP-N3) (see reviews^9^^,^^12^) (Figure 3B). Structurally, the matrix of the mature ZP appears to be organized by ZP2-ZP3 heterodimer filaments cross-linked via ZP1 homodimers.^70^^,^^71^ Although the precise architecture of the ZP has recently been studied in detail,^72^ the specific mechanisms of oligomerization and the exact organization of ZP proteins is not yet fully understood. The postfertilization cleavage of ZP2 is the only known biochemical modification of the ZP by cortical granule content in mammals.^19^^,^^44^

The ZP2 gene is localized on chromosome 7 in mice and on chromosome 12 in humans where it encodes the 713 aa and the 745 aa containing precursor protein.^157^^,^^158^ Primary cleavage by ovastacin is located in the ZP-N2 subdomain at position ^166^LA↓DE^169^ in murine and at ^171^LA↓DD^174^ in human ZP2. This sequence is presumably easily accessible owing to its localization in a flexible loop structure (pdb 5II6 and pdb 8RKE). The ∼120 kDa size of ZP2 is thus processed into a ∼30 kDa N-terminal and ∼90 kDa C-terminal fragment, which remain linked by C^150^-C^255^ (mouse) or C^155^-C^261^ (human) (ZP2_f_).^19^^,^^51^^,^^81^^,^^159^ The overall conservation of the ovastacin-typic acidic cleavage site in mammals and the conserved disulfide bonds in ZP2, linking the fragments, indicate a functional conservation of the cleavage in all mammals (Figure 3B). Noteworthy, even in the African clawed frog (*Xenopus laevis*), ZPA (ZP2 ortholog) is cleaved by a metalloproteinase after fertilization and, like in mammals, triggers envelope hardening.^160^

In mice, the presence of additional peptide fragments with a lower molecular weight indicates secondary cleavages by ovastacin at positions ^125^YK↓DD^128^ and ^52^DK↓DE^55^. This implies that the smaller fragment comprises four different variants.^19^ Structurally, this cleavage triggers a partial refolding and probably induces a conformational change involving ZP-N1 and ZP-N2 of ZP2, which enables ZP2_f_ to oligomerize via ZP-N2 and ZP-N3 subdomains.^72^ These changes suggest a rigid cross-linking of the ZP filaments. This model could explain the physiological changes of the ZP, i.e., mechanical hardening and disruption of sperm binding. Despite other potential modifications of the ZP in the course of egg-sperm interaction and fertilization, only ovastacin is able to completely abrogate sperm binding.^16^^,^^19^

### Fetuin-B and inhibition of ovastacin

Fetuin-B is a liver-derived plasma protein, a member of the cystatin superfamily. This family covers the stefins (type 1 cystatins), the cystatins (type 2 cystatins), and the kininogens and the fetuins (type 3 cystatins).^161^ In general, cystatins are extensively studied competitive inhibitors of cysteine proteases and are, among others, recognized for the regulation of cathepsins.^161^^,^^162^^,^^163^^,^^164^^,^^165^ They feature at least one cysteine-like domain, characterized by a typical fold of five stranded anti-parallel β-sheets wrapped around a central N-terminal helix (pdb 1CEW).^166^ With its sharp edge, the wedge-shaped fold inserts into the active site cleft of the cysteine protease. The binding to the protease is mediated via two β-hairpin loops and the N-terminal trunk, which is why the inhibition mechanism was termed the “elephant trunk model.”^167^ The type 3 cystatins, including fetuin-A (α-2-Heremans-Schmid glycoprotein, *AHSG*, in humans) and fetuin-B, do not exhibit any capacity to inhibit cysteine proteases.^61^^,^^168^ Initially, fetuin-A was described as a specific inhibitor of the astacin proteases meprin α and meprin β.^169^ However, later it became apparent that this inhibition was a result of contamination with fetuin-B.^17^^,^^61^ Owing to their strong biochemical similarity, fetuin-A preparations from serum were frequently contaminated with fetuin-B.

Encoded by the *FETUB/Fetub* gene, located on chromosome 3 in humans and on chromosome 16 in mice,^62^^,^^170^ fetuin-B is expressed almost exclusively by the liver.^62^^,^^63^^,^^170^ The serum concentrations are estimated at 3 μM in mice and 100 nM in humans.^63^ The murine transcript is spliced out of eight exons and encodes the following domains: (1) a signal peptide (SP, 18 aa, pos. 1–18), (2) the two cystatin-like domains CY1 (109 aa, pos. 34–143), (3) the CY2 (107 aa, pos. 159–266), and (4) a C-terminal region (CTR, 18 aa, pos. 270–388) (Figure 3C). CY1 and CY2 are interconnected by a short linker (15 aa, 144–158). The human ortholog comprises 382 aa. Structurally, both cystatin-like domains (CY1 and CY2) feature the characteristic cystatin-like fold. Within these two domains, C^120^-C^140^ and C^237^-C^263^ connect β4 with β5 (CY1) and β9 with β10 (CY2), respectively. The structures that are homologous to the “legumain-binding loop”^171^ are stabilized by C^96^-C^107^ in CY1 and C^217^-C^224^ in CY2, respectively. CY1 is rigidly positioned relative to CY2 via the disulfide linked (C^154^-C^157^) trunk (pdb 6HPV)^17^^,^^172^^,^^173^ (Figure 3C). Despite its largely disordered structure, the CTR appears to be positioned adjacent to CY2, with which it is linked via C^38^-C^374 17^. In several cell culture models, fetuin-B exhibits complex glycosylation.^61^ However, only *N*-linked glycosylation at N^40^ and N^139^ (CY1) has so far been confirmed.^17^^,^^173^

Meanwhile, we recognized that fetuin-B only features inhibitory capacity on certain representatives of the astacins (only non-BTPs), as it has no inhibitory effects toward metalloproteinases in general or on other protease classes such as cysteine, serine, or aspartate proteases.^61^ X-ray crystallography of fetuin-B in complex with astacin and meprin β revealed the precise mechanism of inhibition.^17^^,^^172^^,^^173^ We have previously demonstrated that fetuin-B potently inhibits ovastacin, meprin α, and meprin β in a competitive manner by utilizing its ^154^CPDCP^158^ trunk, which is inserted into the active site cleft.^17^ The sidechain of D^156^ interacts with the catalytic zinc ion, whereas the conserved hairpin I (QWVxGP) of CY2 interacts with the substrate-binding pockets S′2 and S′3 on the non-prime site of the enzyme (Figure 3E). Thereby, the substrate-binding pockets S1 and S1′ are omitted, and the inhibitor is not cleaved. The inhibition constants (*K*_i_) of fetuin-B for different astacins from different species are all in the pico- to low nanomolar range.^17^^,^^21^ Thus, both glycosylation and the CTR have no significant impact on the inhibitory capacity of fetuin-B.^61^^,^^172^ However, specific point mutations, for instance D^156^A, reduce the affinity toward ovastacin by up to four orders of magnitude (*K*_i_ = 0.05 nM [wild-type] vs. 425 nM [D^156^A]).^17^ The overall conservation of the CPDCP in vertebrates emphasizes its importance for inhibition (Figure 3D). Unlike the originally described inhibition of cystatins (“elephant trunk model”), this novel type of inhibition (“raised elephant trunk model”) primarily involves the interaction of the CPDCP trunk and hairpin I of CY2 interacting with the active site of the enzyme.^17^^,^^173^ The inhibition of ZPA cleavage by fetuin in *Xenopus laevis*^160^ suggests a phylogenetic conservation of this mechanism in the context of fertilization and hints toward the ZPA protease being a non-ovastacin orthologous astacin.

To date, only the inhibition of ovastacin has been reported in terms of its organismic function in mammals. It remains open whether the inhibition of the meprins by fetuin-B is of physiological relevance.^173^^,^^174^^,^^175^ Nonetheless, the strong conservation of the structures responsible for inhibition in species that do not yet have ovastacin evolved, as well as the ubiquitous presence in blood plasma, suggest further physiological functions of fetuin-B that would be of relevance for potential future clinical strategies.

## Clinical significance

In recent years, new evidence has emerged emphasizing the crucial role of ovastacin activity in individuals affected by infertility. In this section, we summarize clinically reported types of ovastacin dysregulations and discuss their effects based on the functional and structural context outlined in the previous section.

One study identified a point mutation within the *ASTL* gene in two siblings causing exon 6 skipping,^46^ which is associated with female infertility. This mutation likely leads to a loss of catalytic function as exon 6 encodes crucial structures of the catalytic domain including the zinc-binding motif (see “Biochemical, Structural and Phylogenetic Aspects” section) and thus likely results in an absence of ZP2 cleavage (Figure 3F). Accordingly, a lack of ZP2 cleavage in humans is probably linked to female infertility. Other point mutations in the human *ASTL* gene, leading to single amino acid exchange within the catalytic domain, have also been described to cause female infertility.^94^^,^^144^ Based on the aforementioned key elements involved in the astacin fold, these mutations suggest a significant impairment of the catalytic activity of ovastacin. The H^186^L mutation is located in the zinc-binding consensus motif. The R^117^C mutation within the α-helix A presumably introduces a misplaced disulfide bridging, possibly involving C^419^, and could prevent proper folding. The mutation R^274^W, localizing in the α-helix C, disrupts the ionic interaction with D^271^, which also might be structurally critical.^144^ L^184^H, facing the hydrophobic core of the upper subdomain, could also impair functional fold via introducing steric hindrance (Figure 3F). Transgenic mice carrying these mutations (L^184^H, H^186^L, or R^274^W) do not display ZP2 cleavage, and their fecundity is significantly reduced.^94^ Although the authors of the respective studies observed in mice an increased rate of polyspermy *in vitro*, its physiological occurrence *in vivo* remains unresolved (see “Physiology of ZPH and Regulation of ZP2 cleavage section”).^20^^,^^40^^,^^79^^,^^88^ Likewise, in humans, increased polyspermy has not yet been confirmed as a cause of infertility derived from a loss of ovastacin activity. Furthermore, it is noteworthy that an absence of ZPH in mice appears to be associated with reduced fecundity, whereas in humans it is linked to infertility. As already discussed, the cleavage of ZP2 might have other functions beyond polyspermy prevention, i.e., ensuring successful embryo development up to hatching and implantation.^68^^,^^103^ Considering that moonlighting functions of the ZP proteins have just been discovered,^176^ the ZP2 cleavage might additionally contribute to functions currently unexplored.

Our literature analysis did not reveal any equally well-described mutations in either the genes encoding fetuin-B or ZP2 that are linked to ZPH. Genomic databases and current publications list multiple point mutations in the *ZPA* gene; however, none have been reported in the ZP-N2 module proximal to the ovastacin cleavage site. Nevertheless, several of these published mutations in ZP2—particularly the ones in the C-terminal region—are associated with infertility and implantation failure.^177^^,^^100^^,^^101^^,^^102^^,^^178^^,^^179^ Due to the lack of data regarding their effects on the overall ZP architecture and integrity, the potential impact on ZPH is currently not assessable. To date, the variants and point mutations in fetuin-B deposited in the databases have not been linked to female infertility. However, these mutations are not located in regions crucial for metalloproteinase inhibition (CPDCP trunk and hairpin I of CY2). Nevertheless, given the conservation of CPDCP across all vertebrates (Figure 3D) and its essential requirement for high-affinity inhibition,^17^ it is likely that mutations within this region might contribute to female infertility in humans as well. Collectively, a loss of ovastacin activity or absence of ZPH is directly associated with female infertility in humans. This highlights the clinical significance of ovastacin regulation and its therapeutic potential.

### Options to modulate fertilization via controlling ovastacin activity

As noted above, premature ZPH is a limiting factor for fertilization *in vitro*. Even within the first hour after egg collection, it appears to gradually reduce fertilization success.^98^ This represents a dramatic restriction of the time frame for fertilization compared with *in vivo* conditions. Moreover, IVF failure appears to correlate with fetuin-B plasma levels of oocyte donors in response to hormone treatment.^180^ Already in the early 1990s, studies discovered premature ZPH, and the subsequent reduction of the fertilization rate was prevented by supplementation with serum^181^ or purified fetuin, which contained both fetuin-A and fetuin-B.^99^^,^^170^ Once it was discovered that the absence of fetuin-B in IVF media and insufficient inhibition of ovastacin cause premature ZPH,^16^ supplementation of purified heterologously expressed fetuin-B was introduced. This not only prevented ZPH *in vitro* and increased fertilization success but also extended the time of successful fertilization.^98^ This supplementation might also overcome the clinical need for intracytoplasmic sperm injection (ICSI) in some cases and, at least partially, would enable a physiological selection including a compatibility check. Moreover, it might reduce the number of eggs required for a successful pregnancy compared with classical IVF. Conversely, a proof-of-concept study also demonstrated the potential to interfere in the inhibition of prematurely released ovastacin *in vivo.*^34^ Using antisense oligonucleotides targeting fetuin-B, a temporary downregulation of plasma fetuin-B levels in mice induced a temporary contraceptive effect.

To overcome the various limitations of proteinogenic inhibitors, such as hydrophilicity, costly production, or biological half-life, small molecule compounds were developed for many proteases.^182^^,^^183^^,^^184^^,^^185^ Tertiary-amine-based hydroxamate inhibitors capable of inhibiting ovastacin in nanomolar concentrations have already been reported and may, in future, substitute fetuin-B supplementation *in vitro.*^186^^,^^187^ Even so, the structural similarity between ovastacin and the meprins described above certainly poses a serious challenge for systemic application of such molecules in terms of specificity and off-target effects. However, as the meprins are not expressed by the early embryo, they may become applicable in IVF to substitute fetuin-B to block premature ZPH.

## Future aspects

Ovastacin and fetuin-B orchestrate the properties of the ZP in a fine-tuned interplay and thereby control sperm access to the egg. Both the lack of ovastacin activity as well as excessive ovastacin activity have contraceptive effects, at least in humans. This turns ovastacin into a promising candidate to target both infertility and contraception. New therapeutic approaches targeting ovastacin activity might increase *in vitro* fertilization efficiency. Furthermore, modulation of the ovastacin inhibition by fetuin-B opens avenues for new contraceptive strategies. Small molecule compounds in particular may represent a suitable tool to improve the outcome of ART and to answer the demand for non-hormonal contraception. This is of particular importance not only for humans but also economically in animal reproduction, considering the widespread use of ART including IVF in farm animals, which is now more common than natural conception for many species. Still, for systemic contraceptive application of such compounds, their selectivity will likely pose the major challenge.

## Acknowledgments

We thank Michael Plenikowski for the digital realization of Figures 1 and 2. The authors thank Fazilet Bekbulat and Walter Stöcker for the fruitful discussions and critical reading of the manuscript. This work was supported by a grant from Deutsche Forschungsgemeinschaft (DFG) to H.K. (KO 6071/2-1).

## Author contributions

Conceptualization, N.v.W. and H.K.; literature search, N.v.W. and H.K.; visualization, C.B. and H.K.; writing—original draft, N.v.W. and H.K.; writing—review & editing, N.v.W., C.B., and H.K.; funding acquisition, project administration, and supervision, H.K.

## Declaration of interests

The authors declare no competing interests.
